# Intermolecular Interactions and Spectroscopic Signatures of the Hydrogen-Bonded System—n-Octanol in Experimental and Theoretical Studies

**DOI:** 10.3390/molecules27041225

**Published:** 2022-02-11

**Authors:** Michał Pocheć, Katarzyna M. Krupka, Jarosław J. Panek, Kazimierz Orzechowski, Aneta Jezierska

**Affiliations:** Faculty of Chemistry, University of Wrocław, ul. F. Joliot-Curie 14, 50-383 Wrocław, Poland; 307313@stud.chem.uni.wroc.pl (K.M.K.); jaroslaw.panek@chem.uni.wroc.pl (J.J.P.); kazimierz.orzechowski@chem.uni.wroc.pl (K.O.)

**Keywords:** n-octanol, hydrogen bond, isotope effect, gas phase, liquid phase, FTIR, DFT, PCM, AIM, SAPT, classical MD, CPMD, PIMD, snapshot-envelope

## Abstract

n-Octanol is the object of experimental and theoretical study of spectroscopic signatures and intermolecular interactions. The FTIR measurements were carried out at 293 K for n-octanol and its deuterated form. Special attention was paid to the vibrational features associated with the O-H stretching and the isotope effect. Density Functional Theory (DFT) in its classical formulations was applied to develop static models describing intermolecular hydrogen bond (HB) and isotope effect in the gas phase and using solvent reaction field reproduced by Polarizable Continuum Model (PCM). The Atoms in Molecules (AIM) theory enabled electronic structure and molecular topology study. The Symmetry-Adapted Perturbation Theory (SAPT) was used for energy decomposition in the dimers of n-octanol. Finally, time-evolution methods, namely classical molecular dynamics (MD) and Car-Parrinello Molecular Dynamics (CPMD) were employed to shed light onto dynamical nature of liquid n-octanol with emphasis put on metric and vibrational features. As a reference, CPMD gas phase results were applied. Nuclear quantum effects were included using Path Integral Molecular Dynamics (PIMD) and a posteriori method by solving vibrational Schrödinger equation. The latter applied procedure allowed to study the deuterium isotope effect.

## 1. Introduction

**Figure 1:**
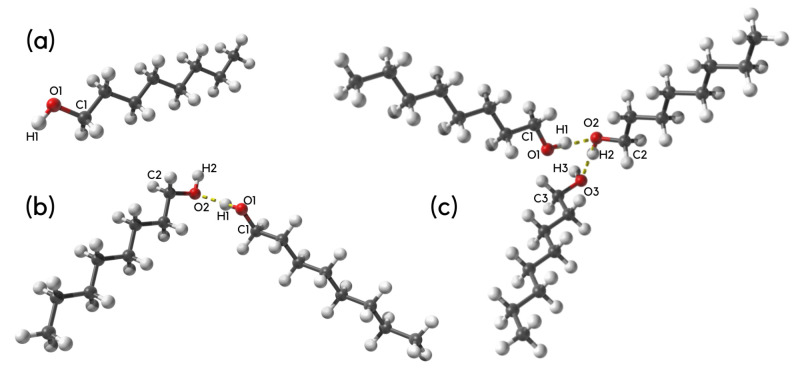
Molecular forms of (**a**) monomer, (**b**) dimer and (**c**) trimer of n-octanol obtained as a result of DFT calculations at the PBE/aug-cc-pVTZ level of theory. Atoms coloring scheme: oxygen atoms—red, hydrogen atoms—white and carbon atoms—grey. The numbering scheme of selected atoms was prepared especially for the current study. The presence of the exemplary intermolecular hydrogen bond is denoted by dotted line.

Alcohols are among the most extensively studied classes of chemical compounds because of their diverse uses [1,2,3,4]. In particular, they have been found as food additives, industrial precursors in many reactions, as well as drugs and drug matrices, e.g., [5,6,7,8]. Linear monohydroxy alcohols are organic molecules containing a hydrogen atom attached directly to an oxygen in the -OH group [9]. Therefore, they are capable of forming hydrogen bonds, both as proton donors and acceptors and become strongly hydrogen-bonded liquids [10]. It is evident in the changes of many physical properties, such as melting point, dielectric constant, and spectral behavior [10,11,12,13]. n-Octanol, aliphatic alcohol containing 8 carbon atoms, was chosen as an object for the current study (see Figure 1). It is not only used as a food additive [7], but it was also investigated as a potential anti-tremor drug [14,15]. It has already been studied via IR spectroscopy in liquid and solid states [16,17], and bears a great similarity (with regard to spectral composition) to n-decanol, studied previously via nonlinear dielectric effect (NDE) and IR spectroscopy [18,19,20]. We have employed experimental and diverse theoretical approaches to investigate n-octanol molecular features with special attention paid to the intermolecular hydrogen bond. Therefore, it is worth to underline that one of the most prominent features of n-octanol and other alcohols is the formation of aggregates via hydrogen bonding [10,21].

The concept of hydrogen bond has been introduced by Latimer and Rodebush in a study on polarity and ionization phenomena. They stated, that water molecules could create aggregates, in which they are interconnected by weak bonds, described in terms of the Lewis theory of valence [22]. Successively, in 1931 the term “hydrogen bond” was used for the first time by Pauling [23]. In his study, he discovered, that a hydrogen located between two different types of atoms binds them with a specific type of electrostatic interaction. However, a prerequisite for its formation is a large difference in the electronegativity of those atoms and hydrogen [23]. After aforementioned discoveries, the characterization and implications contributed to hydrogen bonding attracted interest in the scientific community and have become the subject of an intense discussion, which led to the evolution of hydrogen bond (HB) concept [24,25,26,27,28,29]. According to the latest knowledge, HB can be defined as an attractive interaction between the hydrogen atom from a molecular component X-H, called donor, and the acceptor site, both within the same molecule or between two different molecules [30]. It is assumed, the X and H-atom are covalently bonded, which entails reduction of electron density in the hydrogen atom region. Therefore the hydrogen atom bears a partial positive charge and the X atom—a partial negative charge [31]. The hydrogen donor (X) and acceptor are both strongly electronegative atoms, such as N, S, O and halogens [32,33,34,35,36]. Furthermore, π-electron systems may also serve as proton acceptors in unconventional hydrogen bonds [28,37]. The research on hydrogen bond has developed to embrace a widening array of knowledge about its structure, strength, symmetry and mechanism of formation [26,38,39]. On the basis of those studies it is possible to divide HBs into several types [27,40]. According to the hydrogen bond strength three classes can be distinguished: strong, moderate and weak, with energetic boundaries at about 2 and 15 kcal/mol [41]. Other common type of hydrogen bonding classification is: intramolecular, which is present within a molecule itself and intermolecular, which occurs between two different molecules [42]; symmetric (both donor and acceptor electronegativity are identical) [43] and asymmetric (donor and acceptor electronegativity are distinct from each other) [44]. It has also been postulated, that hydrogen bond can be partially covalent in character [33,45].

The development of various analytical methods allowed structural studies on hydrogen bonds. Among those methods, infrared spectroscopy (IR) has played a crucial role in their accurate identification and characterization [46]. Conventional hydrogen-bonding interactions lead to significant changes in the infrared spectrum, like frequency shifts and changes of intensity for bands related to functional groups directly involved in the hydrogen bond [47]. The range associated with X-H stretching vibrations is the most sensitive to the formation of the hydrogen-bonded bridges [24]. The presence of HB implies an X-H bond lengthening and is associated with the displacement of spectral lines towards longer wavelengths, known as the red shift [48,49,50,51]. However, there also exist unconventional hydrogen bond interactions which shorten X-H bonds, showing the displacement of the spectrum to shorter wavelengths, known as blue shifts [27,28,47]. In the IR spectrum a characteristic absorption band associated with the O-H stretching vibrations is considerably influenced by hydrogen-bonding interactions [10,51]. In the case of no intermolecular interactions, alcohols give an IR spectrum with the aforementioned band narrow and sharp (for details see e.g., [52]). The hydrogen bond between OH groups greatly broadens it. This phenomenon is connected to the arising of aggregates of various shapes and sizes [53,54,55,56]. Furthermore, as the result of weakening of the covalent OH bond, the red shift is observed [51]. Those major changes in IR spectra allow to draw conclusions of intermolecular interactions in linear alcohols.

Here, we have employed IR spectroscopy and diverse theoretical approaches-–Density Functional Theory (DFT) [57,58], classical molecular dynamics (MD) and first principle molecular dynamics (FPMD) methods—to shed light onto intermolecular hydrogen bond properties in n-octanol and its deuterated form. The Atoms in Molecules (AIM) theory [59] was used to study electronic structure and topology of monomer, dimer and trimer. The non-covalent intermolecular interactions and the energy decomposition in dimers was investigated using Symmetry-Adapted Perturbation Theory (SAPT) [60]. The classical molecular dynamics (MD) method was used and followed by further Car-Parrinello Molecular Dynamics (CPMD) simulation in gas and liquid phases [61]. In addition, the influence of the inclusion of nuclear quantum effects on the intermolecular hydrogen bond and the OH stretching was studied using Path Integral Molecular Dynamics (PIMD) [62,63] and so called snapshot-envelope method [64]. The combined study enabled us to achieve the main aims:

(i) experimental FTIR insight into vibrational features of n-octanol—detailed O-H stretching analysis—hydrogen/deuterium (H/D) isotope effect;

(ii) the metric parameters analysis of the intermolecular hydrogen bond in models of dimer, trimer and liquid n-octanol—comparison with available experimental data;

(iii) the electronic structure changes and topological molecular graph introduced by the presence of intermolecular hydrogen bonds based on Atoms in Molecules (AIM) theory [59]—comparison of monomer, dimer and trimer of n-octanol;

(iv) intermolecular forces investigations present in the studied dimers using Symmetry-Adapted Perturbation Theory (SAPT) [60];

(v) intermolecular hydrogen bond protons dynamics in liquid phase on the basis of classical MD and CPMD (gas and liquid phases);

(vi) spectroscopic features analyses reproduced by the Fourier transformation of the time autocorrelation function of atomic velocity using CPMD data;

(vii) the qualitative estimation of the nuclear quantum effects on the bridged proton position in the dimer (on the basis of PIMD in the gas phase);

(viii) a posteriori inclusion of nuclear quantum effects and their influence on the O-H/O-D stretching by solving vibrational Schrödinger equation.

Up to our best knowledge this is the first multi-approach study revealing intermolecular interactions in n-octanol in liquid phase using experimental and theoretical approaches. For the first time, classical MD was applied to melt the solid state n-octanol and further simulate it as a liquid with application of Car-Parrinello Molecular Dynamics (CPMD).

## 2. Materials and Methods

### 2.1. Experimental IR Spectra Measurements

n-Octanol of over 99% purity was used for FTIR spectra measurements, as well as as a substrate for deuterization. The ${}^{1}$H (H) to ${}^{2}$H (D) substitution in the hydroxyl group was performed by vigorous mixing of a two-phase system: n-octanol + deuterium oxide (of over 99% purity) in volume ratio 1:2, in room temperature. After 24 h the obtained phases were separated and the process was repeated with fresh D${}_{2}$O. Deuterization ratio was controlled with IR spectroscopy. Yield of deuterization of the hydroxyl group was estimated to be over 95%, with the CH${}_{X}$ groups remaining undeuterated. For the FTIR experiment samples of n-octanol and deuterated n-octanol were dried using molecular A3 sieves and CaSO${}_{4}$, respectively.

n-Octanol and deuterated n-octanol spectra were acquired using Nicolet Magma 860 FTIR spectrometer. Each spectrum was a result of averaging of 12 scans performed in 4000 to 700 cm${}^{-1}$ range, with resolution of 1 cm${}^{-1}$. Samples were prepared by squeezing a droplet of liquid between two KBr windows. The spectra acquisition was conducted in nitrogen atmosphere. The temperature was controlled using a custom temperature control accessory, with resolution of 0.5 deg. and absolute error of 1 deg. The temperature of spectra acquisition was set to 293 K.

### 2.2. Computational Methodology for the Static DFT and SAPT Models

#### 2.2.1. Static Density Functional Theory (DFT)

The initial models for static quantum-chemical simulations were prepared on the basis of crystal structure of n-octanol deposited in the Cambridge Crystallographic Data Centre (CCDC) [65]. The reference code of the structure in CCDC is 263655 [66]. We have extracted monomer, dimer, trimer (see Figure 1 for details) for further Density Functional Theory (DFT) study [57,58]. The geometry optimization was performed at the PBE/aug-cc-pVTZ level of theory [67,68,69]. In order to confirm that the obtained structures correspond with the minima on the Potential Energy Surface (PES) the harmonic frequencies were computed. No imaginary frequencies were detected. The simulations were carried out in the gas phase and with continuum solvation model (Polarizable Continuum Model—IEF-PCM formalism [70]) using octanol as a solvent. The obtained models were used for structural, electronic structure and spectroscopic analyses with emphasis put on the intermolecular hydrogen bond features. The isotope effect was analyzed as well by exchanging the hydrogen atom from the hydroxyl group to the deuterium. Next, the wavefunctions for Atoms in Molecules (AIM) theory [59] were computed according to the setup described above. The AIM method was applied for electronic structure (atomic charges) and topological study of n-octanol in the gas phase and with solvent reaction field (PCM model with octanol). The electron density and its Laplacian at Bond Critical Points (BCPs) were computed to confirm the presence of hydrogen bonding. The quantum-chemical computations were performed with the Gaussian 16 Rev. C.01 suite of programs [71]. The AIM analysis was carried out with the AIMAll program [72]. The graphical presentation of the obtained results was prepared with assistance of the VMD 1.9.3 [73] and Samson [74] programs.

In the case of DFT calculated spectra, the post processing included scaling of wavenumbers of the absorption maxima (to eliminate the method drag) as well as Gaussian envelope of the bands. This resulted in spectra akin to the real-life shape of bands. The wavenumber scalling factor was set as 0.985, and the Gaussian-envelope width was set as 70 cm${}^{-1}$ for the νOH bands and 8 cm${}^{-1}$ for the rest of the bands. All of the spectra were normalized.

#### 2.2.2. Symmetry-Adapted Perturbation Theory (SAPT)

In the next step the energy decomposition of the dimers of n-octanol was carried out using Symmetry-Adapted Perturbation Theory (SAPT) [60]. The interactions energy was estimated for two sets of structures: (i) extracted from the X-ray experimental data measured at 190 K [66]; (ii) obtained as a result of quantum-chemical simulations at the PBE/aug-cc-pVTZ level of theory in the gas phase [67,68].

The energy decomposition based on SAPT method was performed at the SAPT2 level of theory [75]. The SAPT2 calculations for the experimental and PBE/aug-cc-pVTZ optimized structures were carried out with the aug-cc-pVDZ basis set [69]. The interaction energy computation involved the Basis Set Superposition Error (BSSE) estimation [76], dividing the investigated dimers into “monomers”.

In the SAPT analysis an exact Hamiltonian of the system is divided into the Hartree-Fock descriptions of monomers A and B, ${\hat{F}}_{A}$ and ${\hat{F}}_{B}$, correlation components interacting inside the monomers, ${\hat{W}}_{A}$ and ${\hat{W}}_{B}$, and the part describing interaction between monomers, $\hat{V}$. This gives the formula:

(1) $$\hat{H}={\hat{F}}_{A}+{\hat{F}}_{B}+{\hat{W}}_{A}+{\hat{W}}_{B}+\hat{V}$$

This allows for the use of perturbational expansion with strict enforcement of partitioning between inter- and intra-monomer terms (hence the “Symmetry-Adapted” part of the method name). The resulting partial contributions are very characteristically labelled by a two-number system, e.g., $E_{exch}^{12}$ is a Pauli repulsion (exchange) term of the first order in the intermolecular operator and second order in the intramolecular correlation part. The SAPT terms can be amended by Hartree-Fock $\delta^{HF}$ or MP2 $\delta^{MP2}$ corrections which gather higher-order terms. The form used in this study includes the $\delta^{HF}$ term. The SAPT analysis was conducted using the Psi4 1.2.1 [77] program.

### 2.3. Computational Methodology for the Time-Evolution Study on the Basis of Classical and First-Principle Molecular Dynamics (FPMD)

#### 2.3.1. Classical Molecular Dynamics (MD) Simulations

The liquid phase model of the studied n-octanol was prepared on the basis of the X-ray crystal structure [66]. The Mercury 4.2.0 program [78] was used for the unit cell visualization and super cell construction. As a result, a supercell with 33.65 × 36.29 × 38.94 Å dimension for n-octanol was obtained. Next, the antechamber program, part of the AmberTools21 suite, Ref. [79] was applied to assign force field parameters and create topology for the studied system. Periodic Boundary Conditions (PBCs) were applied to simulate the bulk solution. The GAFF force field [80] was employed for the study. Non-bonded Van der Waals and short range electrostatic interactions were switched off at 10 Å. The particle mesh Ewald (PME) method was applied to evaluate the long range electrostatic interactions [81,82]. The model was prepared with assistance of the TLEAP program [83] implemented in the AmberTools21/AMBER 20 suite of programs [84,85]. First, the energy minimization of 1000 steps was carried out to remove short contacts in the prepared model of n-octanol. Secondly, the investigated system underwent NVT simulations with cell size enlarged to 40 Å, which were taken as equilibration time to melt the solid in order to obtain the liquid phase. The simulation temperature was set to 300 K and controlled by Langevin thermostats [86]. A time-step of 2.0 fs was employed to propagate the equations of motion. The NVT equilibration total simulation time was 100 ns. In the third step, the simulation was switched to NPT ensemble, regulated by Langevin thermostats and barostats set at 300 K and with 1 atm conditions [86,87]—this allowed the system density to equilibrate. The SHAKE algorithm [88] was used to keep at fixed length bonds involving hydrogen atoms. The NPT simulation was also performed for 100 ns. The fourth and final step was the 300 ns production run in the NVT ensemble at 300 K to equilibrate the system at its final density and cell size, a = 39.182 Å. Thus prepared liquid phase model underwent First-Principle Molecular Dynamics simulations (FPMD).

#### 2.3.2. Car-Parrinello Molecular Dynamics (CPMD) in the Gas and Liquid Phases

Car-Parrinello Molecular Dynamics (CPMD) [61] simulations were carried out in vacuo and in the liquid phase of n-octanol. The models for gas phase theoretical studies were prepared on the basis of crystal structure deposit CCDC code 263655 [66]. The monomer, dimer and trimer of n-octanol were extracted from the crystal structure and further used for the gas phase CPMD simulations at 190 K temperature. The computations were performed in orthorhombic boxes with a = 14 Å, b = 16 Å and c = 18 Å for the monomer; a = 16 Å, b = 16 Å and c = 25 Å for the dimer; a = 16 Å, b = 18 Å and c = 26 Å for the trimer with the Hockney periodic image removal scheme applied. The liquid phase model was prepared taking the last frame of the classical molecular dynamics (MD) production run. The computational setup for this part of the simulation was as follows: the Perdew, Burke and Ernzerhof (PBE) functional [67] was employed for the exchange and correlation energy approximation. The norm-conserving Troullier-Martins pseudopotentials [89] were applied to replace the core electrons of the atoms in the studied alcohol. The kinetic energy cutoff of 100 Ry was applied for the plane-wave basis set. The time step was consistently set to 3 a.u. while the fictitious electron mass parameter (EMASS) was equal 400 a.u. The temperature was controlled by Nosé-Hoover thermostat [90,91] and it was set to 300 K in the liquid phase during the simulation time. The computations were performed with periodic boundary conditions (PBCs) and with real-space electrostatic summations for the eight nearest neighbors in each direction (TESR = 8) in the liquid phase. The dispersion corrections by Grimme [92] (Grimme’s DFT-D2 method) were included to reproduce weak interactions. The initial part of the CPMD runs was taken as an equilibration (45,000 steps for the gas phase, 5000 steps for the liquid) and it was not considered during the trajectories analyses. The data for further post-processing were collected for ca. 70 ps for the gas phase and 2.2 ps for the liquid respectively using the CPMD ver.4.3-4610 program [93]. On the basis of the CPMD trajectories the time-evolution analyses of metric parameters of intermolecular hydrogen bonds were performed. Spectroscopic properties of the n-octanol gas and liquid phases were studied based on atomic velocity collected during the production run. The Fourier transformation of the autocorrelation function was employed to obtain the power infrared (IR) spectra. The CPMD results were analyzed with assistance of the following approaches: the intermolecular hydrogen bonds dynamics was visualized and inspected using scripts available in the VMD 1.9.3 suite of programs [73]. The Fourier transform power spectrum of atomic velocity was computed using home-made scripts.

#### 2.3.3. Path Integral Molecular Dynamics (PIMD) in the Gas Phase

Path Integral Molecular Dynamics (PIMD) [62,63] simulations were carried out for the dimer of n-octanol with intermolecular hydrogen bond. The model was prepared using the X-ray crystal structure of n-octanol reported by Shallard-Brown et. al. [66]. For the computations, a similar setup to the one applied for the CPMD was used. The cubic cell with a = 25 Å was used during the PIMD run. The calculations were carried out at 190 K. For imaginary time path integration eight Trotter replicas (P = 8) were applied. The data were collected for 16.7 ps after the initial equilibration of 45,000 steps. The obtained data served to indicate the proton position in the intermolecular hydrogen bond. The PIMD simulations were performed with assistance of the CPMD ver. 4.3-4610 program [93].

#### 2.3.4. A Posteriori Inclusion of Quantum Effects

The nuclear effects of proton motion were included on the basis of a consistent approach using Car-Parrinello Molecular Dynamics (CPMD) for potential energy surface (PES) sampling and subsequent a posteriori nuclear motion corrections by solving the vibrational Schrödinger equation. One can find the idea and details of the method in [64]. The CPMD trajectory of the n-octanol trimer was sampled at 1.45 ps intervals. The obtained fifty snapshots of the n-octanol trimer trajectory were used as the starting points for the one-dimensional proton potential functions generation for the bridged hydrogen motion from the donor (O2) to the acceptor (O3) atom. The rigid scan method was applied for this purpose. Then the potential function was used for the solution of the vibrational Schrödinger equation to give the vibrational energy levels [94]. The set of obtained vibrational frequencies was further used to define the envelope of the OH stretching mode. Additionally, the deuterium isotope effect was calculated as well [95,96]. The described above procedure of one-dimensional nuclear motion quantization was repeated with the particle mass adjusted for deuterium. Therefore, both O-H and O-D snapshot-envelopes were computed. The CPMD simulations were carried out using the CPMD ver. 4.3-4610 program [93]. The VMD 1.9.3. [73] and Gnuplot [97] programs were used for visualization and graphical presentation of the results.

## 3. Results and Discussion

### 3.1. Experimental IR Data and Static Based DFT Models Describing Spectroscopic Properties of n-Octanol

**Figure 2:**
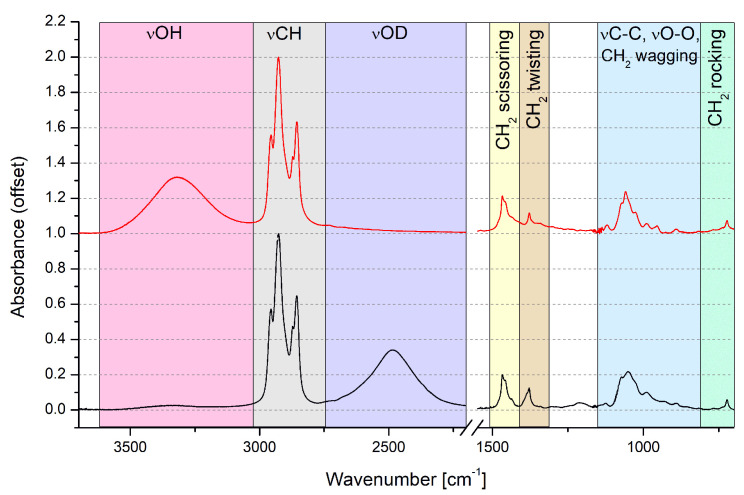
Experimental FTIR spectra of liquid n-octanol (**top**) and deuterated n-octanol (**bottom**). The 2200–1550 cm${}^{-1}$ region was omitted due to the lack of significant bands.

Experimental spectra of liquid n-octanol and deuterated n-octanol are presented in Figure 2. All of the spectral regions have been assigned to specific vibrations using the literature data [16,17,98,99,100,101]. The νOH vibrations have been assigned to very wide, medium intensity band at ca. 3320 cm${}^{-1}$. The CH${}_{2}$ and the terminal CH${}_{3}$ groups νCH vibrations result in the series of bands in the 2800–2980 cm${}^{-1}$ range. The bands: 1467 cm${}^{-1}$ for the scissoring deformation, 1378 cm${}^{-1}$ for the twisting and ca. 710–750 cm${}^{-1}$ for the rocking vibrations are also coming from those groups. The 960–1070 cm${}^{-1}$ broad region comes from the chain C-O and C-C stretching vibrations.

There are large similarities between FTIR spectra of higher, monohydric alcohols in the liquid phase. In n-heptanol, n-octanol, n-nonanol and n-decanol specific spectral regions are analogical [20,102]. This is especially interesting in the light of major differences between closest neighbors, n-nonanol and n-decanol, in the solid state spectra [20]. Most notable of said differences is the νOH region composition—n-nonanol has two, wide bands while n-decanol two sharp bands and an underlying, wide band. In the liquid, however, both of those compounds bear great resemblance to n-octanol. This means, that there is an underlying, equalizing factor for the mid-chain aliphatic alcohols. It should also be noted, that the shortest monohydric alcohols differ in spectral composition from the longer ones.

All of the bands from the assignment have been compared with the most notable results of the DFT calculations for the n-octanol dimer in the solvent reaction field reproduced by octanol as a solvent and the PCM. The comparison is presented in Table 1. Additionally, the results for the monomer and trimer in the gas phase and in the PCM, as well as dimer in the gas phase, have been presented in the Supplementary Material as Figures S2–S4.

When comparing the experimental and theoretical data, the displacement from the computational method is clearly visible—most bands are more than 50 cm${}^{-1}$ higher in DFT compared to the experiment. This a typical behaviour, and is the innate property of the chosen theoretical method using harmonic approximation. The only band not visible in the IR experiment is the free OH stretching vibrations band. This is due to the n-octanol forming aggregates in the liquid state, where every OH group is hydrogen bonded [103,104]. It has been postulated, that this alcohol may preferentially exist as ring tetramers and trimers in pure liquids [104,105].

The dimer in the PCM was chosen as a comparison because it contains the hydrogen bond between the OH groups and both chains are fairly similar, what resembles the real-life system. The monomer lacks the H-bonding, and in the trimer one of the chains has a larger vibrational freedom than others due to the spatial distribution of the chains. This results in splitting of the bands.

**Figure 3:**
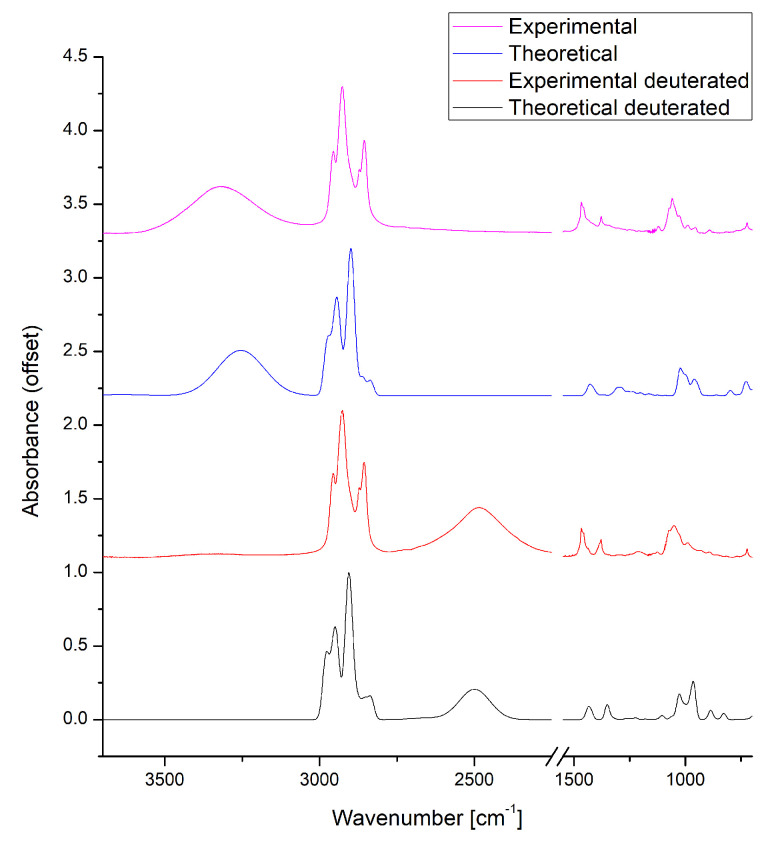
Experimental and theoretical IR spectra of n-octanol. The 2550–1550 cm${}^{-1}$ region was omitted due to the lack of significant bands. The theoretical spectra were computed at the PBE/aug-cc-pVTZ level of theory using PCM model to reproduce the solvent reaction field.

For experimental and DFT methods, the deuterated octanol was also studied. The main difference is the location of the broad stretching band of the hydroxyl group. In the O${}^{1}$H isotopologue (OH) the band is located above the νCH region, around 3300 cm${}^{-1}$, while in the O${}^{2}$H (OD)- below the νCH region, closer to 2600 cm${}^{-1}$. The spectral comparison between isotopologues in both methods is presented in Figure 3. To accommodate for the method systematic shift, the wavenumber scaling factor was introduced. In the case of presented calculated spectra the scaling factor for both H and D isotopolgues was estimated as 0.985.

It should be noted, that the νOH band shift from hydrogenated to deuterated hydroxyl group form is consistent with the literature announcements [106,107]. The νOD is narrower and slightly asymmetrical, with steeper slope in the lower wavenumbers. The remaining parts of the spectrum are almost identical between the isotopologues, with the exception of a medium band around 1050 cm${}^{-1}$, where one of the bending vibrations bands of OH/OD is located. Calculated intensities and locations of the bands for all of the studied systems are presented in Supplementary Material as Table S3. Increase in intensity is one the signs of presence of hydrogen bonding between molecules. In this study free OH bands rarely exceed 50 km/mol, while H-bonded bands are never lower than 400 km/mol, sometimes exceeding 1000 km/mol.

### 3.2. Molecular and Electronic Structure of n-Octanol Associates: A DFT and Atoms-in-MOLECULES Study

Selected metric parameters of n-octanol are presented in Tables S1 and S2 in the Supplementary Material (for details and the atom numbering scheme see Figure 1 and Figure S1). Special attention was paid to the exemplary intermolecular hydrogen bond in dimer and trimer. The presented results were obtained in vacuo and using implicit solvation model. The X-ray crystallographic data [66] show that the intermolecular hydrogen bond is classified as middle strong. The computed results were compared with crystallographic metric parameters. As it is shown, depending on the structure composition (monomer, dimer and trimer) the computed metric parameters differ. On the overall, however, a good agreement between experimental and computed data was found. It is also important to underline that such a comparison showed the applied theoretical method as able to reproduce properly the molecular structure of the n-octanol.

**Figure 4:**
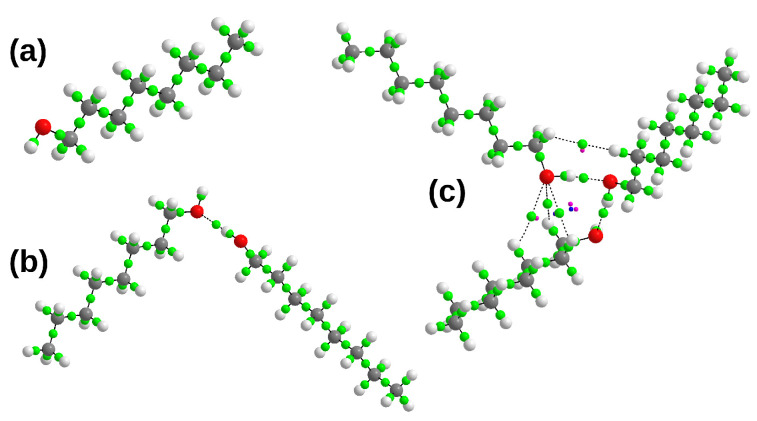
AIM topological molecular graph for the (**a**) monomer, (**b**) dimer and (**c**) trimer of n-octanol. The model was obtained on the basis of geometry optimized at the PBE/aug-cc-pVTZ level of theory in the gas phase. Small green spheres denote Bond Critical Points (BCPs), small pink spheres denote Ring Critical Points (RCPs) wile small blue spheres denote Cage Critical Points (CCPs). Atom color coding:oxygen atom—red, hydrogen atoms—white and carbon atoms—grey.

The electronic structure and topological analyses were carried out using AIM theory [59], for monomer, dimer and trimer of n-octanol. The AIM topological graphs are presented in Figure 4. The analysis confirmed the chemical composition of the monomeric form of n-octanol. In Figure 4a the monomer of n-octanol is presented with green spheres indicating the BCPs. The BCPs are related to the covalent bonding present in the molecule. In the case of the dimer, the AIM topological analysis confirmed the presence of the intermolecular hydrogen bond by locating the BCPs belonging to the OH and H…O bonds. The situation with the trimer of n-octanol varies from that of the monomer and dimer. The AIM analysis indicated the presence of the BCPs associated with the intra- and intermolecular interactions. Beside the covalent bonds, intermolecular hydrogen bonds presence was confirmed. However, as it is shown in the Figure 4c there were detected other interactions e.g., C-H…O and C-H…H-C stabilizing the conformation of the trimer. Due to the formation of additional intermolecular interaction RCPs (red spheres) and CCP (blue sphere) were detected. On the basis of presented data we could speculate that such interactions are present in the liquid and crystal phases of n-octanol being responsible for the spatial arrangement of the molecules.

In the Table 2 net atomic charge values for selected atoms of the discussed structures are presented. In the case of dimer and trimer, atoms involved in the intermolecular hydrogen bonds were taken into account. The atomic charges were computed in the gas phase as well as in octanol as solvent using the PCM. The AIM analysis revealed quantitative differences concerning the electron density distribution depending on the studied structure. In the monomeric form the net atomic charge values slightly differ between the gas phase and solvent reaction field. The difference is 0.0014 for H1, 0.0059 for O1 and 0.0063 for the C1 atom. Moreover, in the gas phase the atomic charges of H1 and C1 are larger than in the octanol environment. The situation is opposite for the O1 net atomic charge - its value decreased in the gas phase. Concerning the dimer, the O1-H1 is a proton-donor group and O2 atom is a proton acceptor in the intermolecular hydrogen bridge. The net atomic charge value of the H1 bridged proton is 0.5834 [e] in the gas phase and 0.5845 [e] in the octanol reproduced by PCM. The difference is equal 0.0011 [e] showing that the environmental influence on the electron density distribution is subtle. The net atomic charge value of the O1 atom decreased comparing with the PCM result. In the case of the proton acceptor - O2 atom the introduction of the octanol increased the net atomic charge value comparing to the gas phase. In the trimer, there are two intermolecular hydrogen bonds formed by O1-H1…O2 and O2-H2…O3 atoms. It is worth to underline that the O2 atom plays in one case a proton-acceptor role, but in the other—its associated O2-H2 hydroxyl group serves as a proton donor. In the case of the H1 bridged proton the net atomic values are 0.5922 [e] in the gas phase and 0.5944 [e] in octanol respectively. Concerning the second bridged proton, H2 atom, the net atomic charge values are 0.5982 [e] in the gas phase and 0.5958 [e] in octanol. In both cases the presence of the octanol as a solvent increased the value of the net atomic charge. Concerning the net atomic charge value of O1, O2 and O3 atoms, in all cases the gas phase value is lower than that obtained upon the solvation. Generally speaking, there is a difference between the values of the net atomic charges obtained as a result of the gas phase simulation and with the implicit solvation model, showing that the solvent can modulate the molecular features.

In Table 3 the values of the electron density and its Laplacian at BCPs are presented. The data were collected for the n-octanol dimer and trimer respectively. The results are strictly associated with the intermolecular hydrogen bonds. The positive values of the electron density and its Laplacian at BCPs H1…O2 in the dimer and H1…O2 and H2…O3 in the trimer confirmed the presence of the intermolecular hydrogen bonds [108]. There is a difference in the electron density and its Laplacian values comparing the gas phase results with the implicit solvation model. In the case of the C1-O1 BCP the difference is 0.0021 for the electron density and 0.0029 for its Laplacian. For the O1-H1 BCP there is 0.0045 for the electron density and 0.0501 for its Laplacian. Concerning the intermolecular hydrogen bond it was found 0.0041 for the electron density and 0.0064 for its Laplacian. The smallest differences were noticed for the O2-C2 BCP—the difference is 0.0012 for the electron density and 0.0001 for its Laplacian. In the trimer, the electron density and its Laplacian at BCPs are 0.0373 and 0.0882 for the H1…O2 and 0.0384 and 0.0884 for H2…O3 intermolecular hydrogen bonds in the gas phase. The introduction of the solvent reaction field resulted in 0.0383 and 0.0897 for the H1…O2 and 0.0392 and 0.0904 for the H2…O3 of electron density and its Laplacian at BCPs respectively. We have estimated qualitatively the electron density and its Laplacian differences upon the implicit solvation. They are: 0.0001 and 0.0223 for the C1-O1 BCP, 0.0018 and 0.0183 for the O1-H1 BCP, 0.001 0.0015 for the H1…O2, 0.0027 and 0.0216 for the O2-C2 BCP, 0.0008 and 0.0020 for the H2…O3 BCP and 0.0027 and 0.0132 for the O3-C3 BCP. These are small differences between electron density and its Laplacian in the intermolecular hydrogen bonds. The electron density increased in the H2…O3 intermolecular hydrogen bond in both phases comparing to the H1…O2. The values of the electron density at the hydrogen bond BCPs are, with quite systematic character, ten times smaller than the electron density at the corresponding covalent O-H bond between the donor and hydrogen atoms. This results indicates some degree of covalency in hydrogen bonding, in agreement with concepts presented in the literature [33,45]. The electronic structure and topological analyses based on the AIM enabled qualitative and quantitative description of intermolecular hydrogen bonding in the n-octanol dimer and trimer. The AIM allowed also the observation of intermolecular interactions of two types: hydrogen bonding between hydroxyl groups in both dimer and trimer, and an additional C-H-O and C-H-H-C interactions, resulting in additional stabilizing factor in trimer.

### 3.3. Origins of Interaction in n-Octanol Dimers—A Symmetry-Adapted Perturbation Theory Investigation

**Figure 5:**
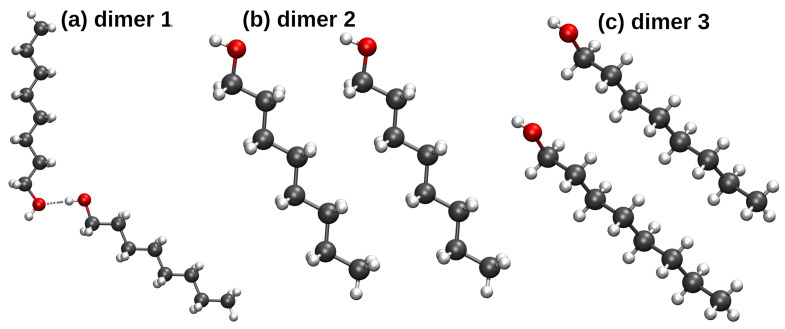
Dimers taken into account in the SAPT study: (**a**) hydrogen-bonded dimer 1, (**b**) dimer 2: molecule 1 above molecule 2, (**c**) dimer 3: molecules aligned alongside. On the basis of the n-octanol X-ray structure [66]. Atom color coding: oxygen atom—red, hydrogen atoms—white and carbon atoms—grey.

The systems selected for the Symmetry-Adapted Perturbation Theory (SAPT) study are designed to reveal the relationship between two possible sources of interaction: hydrogen bonding (dimer 1) and hydrophobic, Van der Waals attraction between the aliphatic chains (dimers 2 and 3)—see Figure 5. The difference between dimers 2 and 3 is the relative position of the hydroxyl groups and chains: dimer 2 has molecule 1 over molecule 2, while dimer 3 has molecule 1 alongside molecule 2.

The results of the SAPT calculations, presented in Table 4, indicate that the three considered dimers are stable and the interaction energies are comparable, although their origin varies between the cases. The hydrogen-bonded dimer 1 possesses large electrostatic contribution, coming from the permanent electronic multipole moments of the monomers. The most important of these, the dipole moments, are aligned at an angle close to 120${}^{\circ }$—less than for the optimal antiparallel arrangement, but favored because of the hydrogen bonding. Moreover, the induction term is also important for the dimer 1, which is characteristic for hydrogen-bonded systems. These two factors, electrostatics and especially induction, are much less pronounced for the dimers 2 and 3, which are instead held by the dispersion forces. This analysis is valid for both sets of structures: taken directly from the X-ray experiment, and optimized at the DFT level. The DFT optimization puts more emphasis on the hydrogen bonding, therefore the related terms—electrostatics and induction—are larger in magnitude for the DFT-optimized dimer 1 than for the corresponding X-ray model. This, however, has also disadvantage of increased mutual penetration of the electronic densities of the two monomers, which results in a much larger exchange term (manifesting itself as Pauli—steric—repulsion).

Summarizing the SAPT study, we wish to note that n-octanol has peculiar property of balance between two different types of interaction. Its aliphatic tail is long enough to provide significant dispersion interactions with similar hydrophobic neighbours. Moreover, the strength of these interactions is comparable to that provided by directional hydrogen bonding between the hydroxyl groups. Dispersion is an important factor also in the case of hydrogen-bonded dimer 1, which is a general property of hydrogen bonding. In this case, the electrostatic—exchange—dispersion ratio indicates the underlying nature of the noncovalent interaction. In the alcohols with much shorter chains (e.g., ethanol, propanol) the hydrogen bonding network dominates, and higher alcohols will be dominated by dispersion. This balance of interactions in the case of n-octanol might be used in rationalization of molecular properties, useful in the design of new systems related to the materials chemistry.

### 3.4. Classical Molecular Dynamics Study of Liquid n-Octanol

Contemporary classical force fields are indispensable tools in simulations of disordered phases, such as liquids. Earlier sections of the current study have shown that association of n-octanol molecules due to hydrogen bonding is responsible for the νOH experimental band shape. While this feature can be analyzed only with reference to the electronic structure methods, structural information on the molecular association can be easily retrieved from the classical MD.

**Figure 6:**
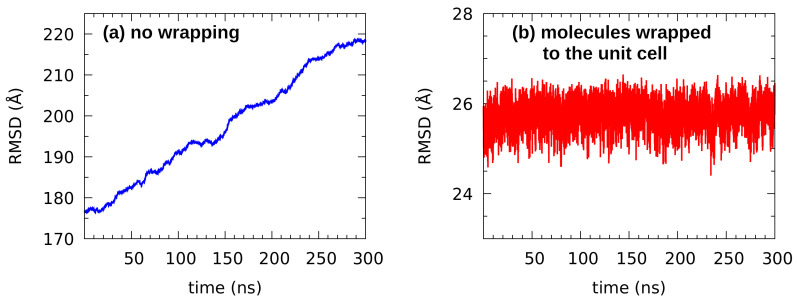
Root mean square deviation (RMSD) from the initial structure for the classical MD simulation of n-octanol.

Root mean square displacement (RMSD) from the reference structure is an important structural parameter summarizing the evolution of the simulated system. In the case of our classical MD study, the reference structure was the initial set of coordinates, directly from the solid state X-ray data [66]. Time course of RMSD, shown in Figure 6a, was prepared without wrapping the molecules back to the unit cell. The resulting graph with quickly growing RMSD indicates that during the production run the molecules drifted freely away from their original positions. On the other hand, the Figure 6b, prepared with the *autoimage* function of the AMBER trajectory analysis module, shows that wrapping the molecules back to the unit cell results in the RMSD oscillating around 25.5 Å with less than 2 Å amplitude. This is characteristic for equilibrated systems, where no large structural events occur—and this is also an important indicator that the strict structural ordering (present in the solid state) has vanished. In support of the claim that the modelled system reached an equilibrium stage, we report that the second equlibration phase, the NPT run of 100 ns at 300 K and 1 atm, rendered the density of the sample as 0.8096±0.0026 g cm^−3^. This result is in a very good agreement with the experimental density of n-octanol at 298 K, 0.8262 g cm^−3^[109], and with earlier simulations with OPLS and GROMOS force fields [110], where density of neat n-octanol spanned the range from 0.8052 to 0.8522 g cm^−3^ depending on the particular force field.

**Figure 7:**
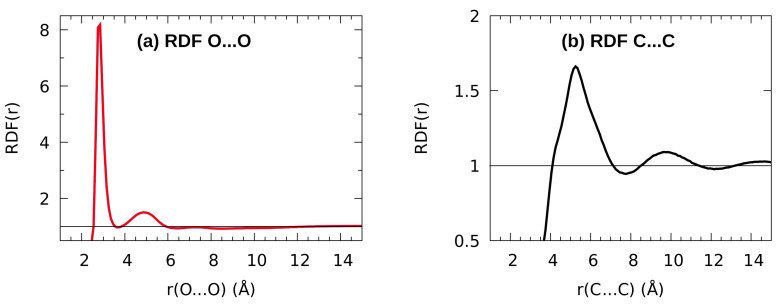
Radial distribution functions (RDFs) for (**a**) hydroxyl oxygen O…O contacts, (**b**) terminal C…C contacts. Results of the n-octanol classical MD simulation.

One of the questions related to the structure of liquids is the presence of local ordering, as opposed to the long-range structural order in the solid state. The best parameter to describe local structural correlations is radial distribution function (RDF). The RDFs for hydroxyl oxygen O…O atom pairs and terminal C-C carbon atom pairs are shown in Figure 7. A strong tendency of n-octanol to form hydrogen-bonded associates is inferred from the presence of a sharp maximum of the O…O RDF at 2.8 Å—a direct O…O interaction (Figure 7a). An integration of the RDF in the range 2.5–3.5 Å yields value of two neighboring molecules: the prevailing local structural motif is that of a hydroxyl group being both a donor and an acceptor of a single hydrogen bond. The ordering is, however, very local, since already the next, second, coordination sphere yields only a broad, low feature spanning from 4 to 5.7 Å, and there are only traces of any variations of the RDF at larger distances. The O…O RDF agrees very well with the GROMOS force field simulation of MacCallum and Tieleman [110]. The long aliphatic chains are conformationally flexible, which is seen in Figure 7b: the terminal C-C RDF has maximum at 5.1 Å, but this feature is very broad, from 4 to 7 Å. This preservation of hydrogen bonding network and loss of conformational ordering are in agreement with the hierarchy and nature of interactions. We have shown in the Section 3.3 that hydrogen bonding and dispersion yield similar contributions to the structural stability of n-octanol, but the discussion of the RDF graphs in the current section points out that the ordering governed by dispersion—a non-directional attraction—is not conserved in the liquid phase, as shown in the broadness of the terminal C-C RDF features.

The last part of the classical MD analysis is an estimation of the lifetime of the hydrogen-bonded associates. Their dynamical nature is evident because of the RMSD fluctuations—the structure is evolving—but strong local ordering is reflected in the RDFs. The best model to combine these two observations is the assumption that the hydrogen bonds continually break and form again, in such a manner that, at a given time, a selected hydroxyl group is saturated with hydrogen bonds (two, according to the RDF profile), but relatively quickly the actual donor and acceptor molecules can change due to the diffusion of molecules in the liquid. Our analysis of the classical MD trajectory revealed that the longest uninterrupted lifetime of a hydrogen bond in n-octanol is 0.8 ns, but there are periods lasting 2.5 ns consisting of several bridge formation—breakup events. These values are much longer than the relaxation times reported for water, where fast librational modes (shorter than 200 fs) were observed without destroying the HB network, while complete reorientation of a water molecule took ca. 2.5 ps [111]; on the other hand, these are maximal values of the residence time registered in the MD production run. Thus, the relaxation and reorientation times in n-octanol can be much larger than in water due to the size of the molecule and to the dispersion forces holding together the aliphatic chain and hampering the conformational dynamics.

### 3.5. Car-Parrinello Molecular Dynamics Analysis of Intermolecular Hydrogen Bonding Network in n-Octanol

The advantage of first-principles molecular dynamics schemes over classical MD is the ability to describe bond breaking and formation, thus chemical reactivity. Moreover, DFT description of hydrogen bonding network is overall much more reliable than within classical force fields. On the other hand, the available time scale is much shorter (routinely less than a nanosecond) than within a classical MD. Fortunately, many dynamical processes connected with hydrogen bonding are fast and can be efficiently investigated using the first-principles MD schemes, such as Car-Parrinello MD [61] used in this study. At this point it is necessary to underline that the CPMD simulations were carried out in two temperatures: 190 K for the gas phase models (monomer, dimer and trimer) to follow the experimental X-ray conditions, and 300 K for the liquid state to avoid spurious freezing phenomena.

**Figure 8:**
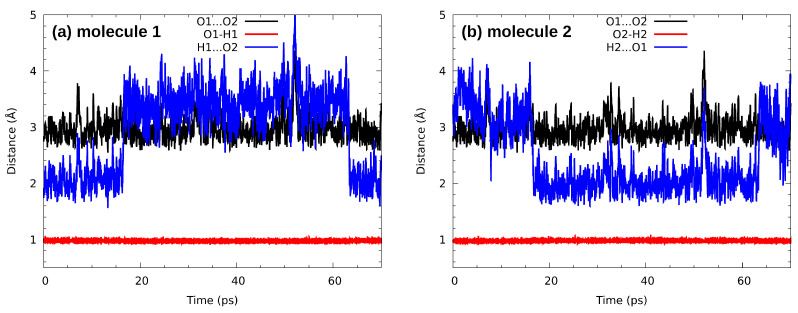
Time evolution of the metric parameters for the intermolecular hydrogen bonds formed by the hydroxyl group from (**a**) molecule 1, (**b**) molecule 2—results of CPMD simulation of the n-octanol dimer in the gas phase.

**Figure 9:**
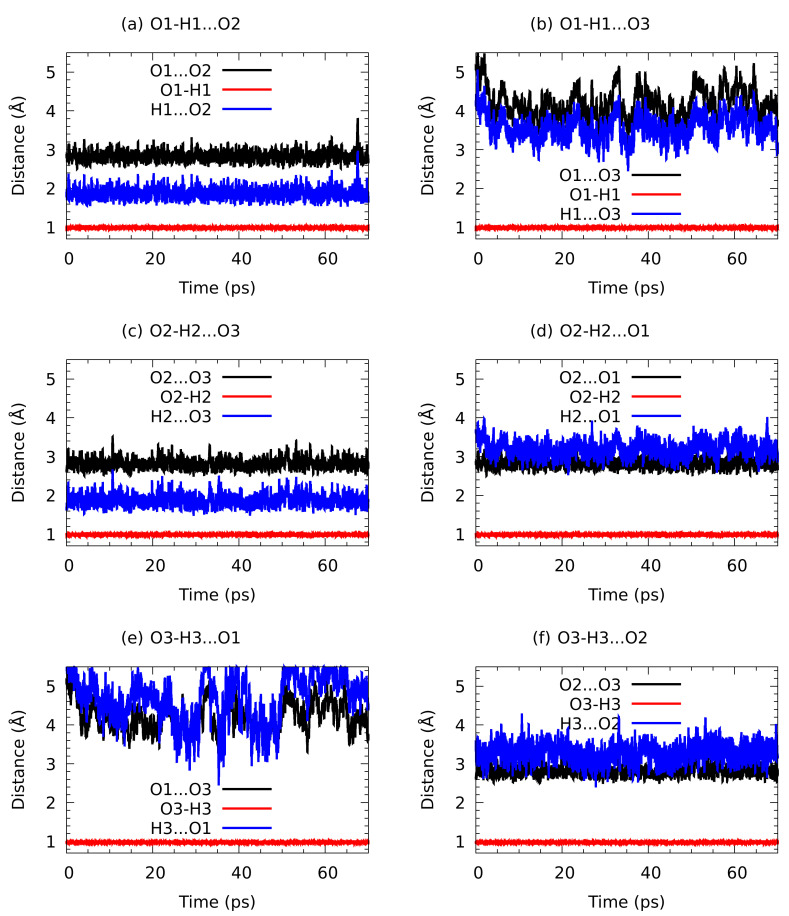
Time evolution of the metric parameters for the intermolecular distances formed between the indicated hydroxyl groups (donor-acceptor): (**a**) molecules 1-2, (**b**) molecules 1-3, (**c**) molecules 2-3, (**d**) molecules 2-1, (**e**) molecules 3-1, (**f**) molecules 3-2. Results of CPMD simulation of the n-octanol trimer in the gas phase.

The analysis of the hydrogen bond metric parameters for the n-octanol dimer in the CPMD gas phase simulation (see Figure 8 for the time evolution) revealed that the O1…O2 bond is stable. The donor–acceptor distance is oscillating around ca. 3 Å. An interesting phenomenon is encountered at 15 and 64 ps of the simulation time. The rotation of the hydroxyl groups changes the roles of donor and acceptor atoms: first the O1-H1…O2 bond becomes O2-H2…O1, and then the situation switches back to the initial configuration. We can expect from the dynamical behaviour of the molecules that the situation repeats. An application of the CPMD method shed more light onto the molecular mechanisms responsible for the intermolecular hydrogen bond dynamics and associated with it molecular features. In Figure 9 the time evolution of interatomic distances of the trimer is presented. As it is shown, the O…O interatomic distances have not changed much in the case of (a), (c), (d) and (f). They oscillate around ca. 3 Å. The exception are (b) and (e) where the O…O interatomic distance strongly fluctuates and averaging it is equal ca. 4.5 Å. The O-H covalent bond during the simulation time has been equal ca. 1 Å in all discussed cases. Concerning the H…O intermolecular hydrogen bond, it oscillates around ca. 2 Å in the case of (a) and (c). It has been elongated up to ca. 3.2 Å for the (d) and (f). Strong fluctuations of the H…O interatomic distance have been noticed for (b) and (e). It oscillates around ca. 3.8 Å in O1-H1…O3 and 4.8 Å in O3-H3…O1. According to the geometric parameters criteria, the H…Y distance should be in the range between 1.60 Å and 2 Å [112]. Therefore, in the case of (b) and (e) it is more suitable to discuss the interatomic interaction rather than HB. However, the presence of such interactions is an additional factor in the spatial structure stabilization. The proton sharing or proton transfer phenomena have not been observed during the simulation time.

The hydrogen bonding network in the liquid n-octanol was also studied with the CPMD approach. This simulation was technically demanding because of the system size, both in terms of the cell dimensions (a = 39.182 Å, related to the plane wave basis set size) and the number of molecules (224) and atoms (6048). The length of the simulation was determined by these factors, and the simulation itself would not be possible without the most recent supercomputing resources.

**Figure 10:**
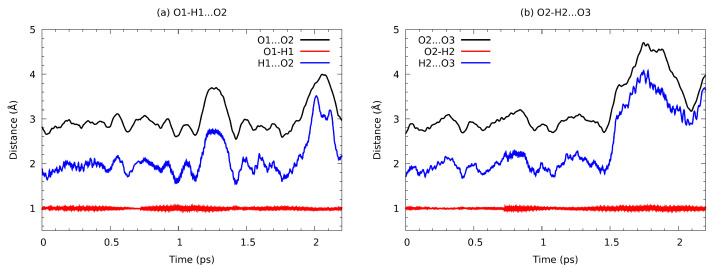
Time evolution of the metric parameters for the intermolecular hydrogen bridges formed between three selected consecutive molecules—results of CPMD simulation of the n-octanol in the liquid phase.

During the liquid phase trajectory analysis, we have selected a random molecule forming hydrogen bonds with its neighbours, in a manner locally resembling the gas-phase trimer of Figure 1. The collected trajectory, limited by the system size (224 molecules with long aliphatic chains), is presented in Figure 10. Even at the picosecond time scale some fast molecular reorientation events are possible. After ca. 1.1 ps simulation time, the O1-H1…O2 bond undergoes temporary elongation, which might in turn add to the disruption of the O2-H2…O3 bond, which starts at 1.5 ps of simulation. The former bond seems to regain its stability, at least for a short period. Such events, as already noted at the end of the Section 3.4, happen at the timescale of a few picoseconds for smaller molecules such as water [111]. Here we observe hydrogen bonding network dynamics only, and the long aliphatic chains slow down the overall progress of dynamical properties.

**Figure 11:**
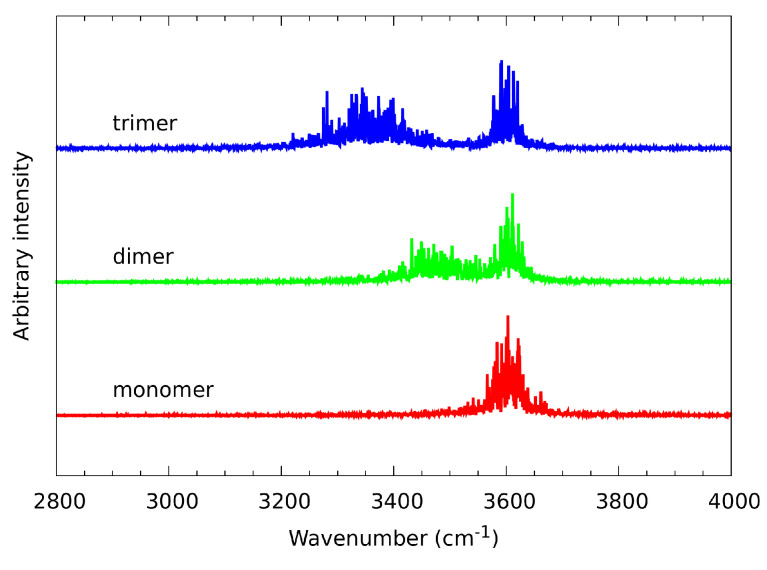
Vibrational signatures (atomic velocity power spectra) of the hydroxyl O-H stretching-results of CPMD simulation of the n-octanol monomer, dimer and trimer in the gas phase.

Analysis of the atomic velocity power spectra is a well-established technique which allows separation of the vibrational features, resulting from the CPMD simulation, into individual group contributions. The Figure 11 shows vibrational stretching features of hydroxyl O-H, derived (using aforementioned technique) from the gas phase CPMD runs for n-octanol monomer, dimer and trimer. The hydroxyl O-H stretching in the monomer was found between 3500 cm${}^{-1}$–3700 cm${}^{-1}$. The gas phase CPMD results enabled a description of the spectroscopic signatures of the OH free group and it could serve as a reference to monitor spectroscopic changes introduced by the presence of the intermolecular hydrogen bond. In dimer, there is a broad absorption region from 3350 cm${}^{-1}$ to 3700 cm${}^{-1}$. The region could be divided into two bands. They differ in intensity and width, but they are not entirely separated. According to the analysis, this is due to the presence of both free as well as hydrogen bonded OH. In trimer, the CPMD results revealed the absorption regions located between 3225 cm${}^{-1}$–3475 cm${}^{-1}$ and 3500 cm${}^{-1}$–3700 cm${}^{-1}$. They are clearly separated, providing information of both hydroxyl protons involved in the intermolecular hydrogen bonds.

In the Figure 11 it is clearly demonstrated that the absorption band between 3500 cm${}^{-1}$-3700 cm${}^{-1}$ is present in each of the studied gas phase cases giving an insight into spectroscopic features exhibited by the n-octanol. This band, corresponding to the free, non-bridged hydroxyl νOH, does not change its position. On the other hand, there is large difference between the positions of the bridged νOH in the dimer and trimer. This is the manifestation of the different vibrational freedom of those systems. The red shift, characteristic to the hydrogen bond formation, is slightly bigger in trimer than in dimer, probably due to the additional spatial restriction introduced by the bigger system, resulting in smaller vibrational freedom.

When comparing experimental and gas-phase spectra it is evident, that only the wide band is visible in both spectra in the 3000–3700 cm${}^{-1}$ region. This means that only the multi-species hydrogen bonded hydroxyl systems are present, with no trace of sharp bands at higher wavenumbers, associated with free OH groups. Also the locations and widths of the bands stand in agreement between both methods. This proves that molecular behaviors observed in CPMD translate well to the behavior of molecules in experimental systems—the temporary, very short-lived presence of free –OH groups cannot be excluded, but on average the molecules have well-defined coordination spheres.

### 3.6. Path Integral Molecular Dynamics Results of the Intermolecular Hydrogen Bonding in the n-Octanol Dimer

**Figure 12:**
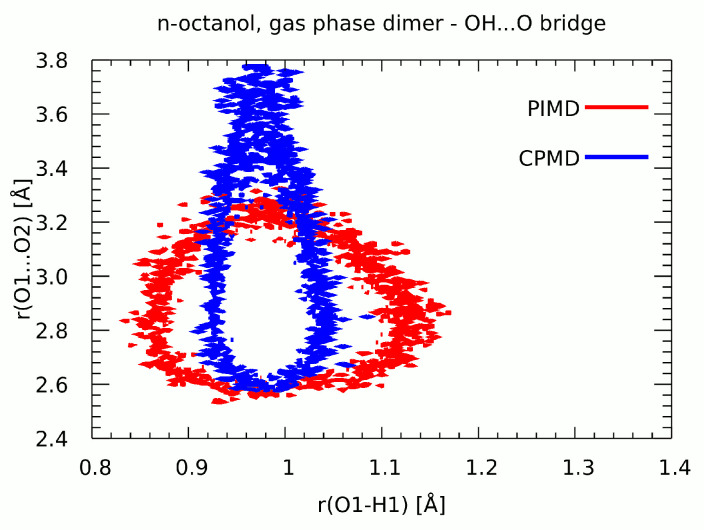
Two-dimensional histograms of the donor-proton and donor-acceptor distances of the O1-H1…O2 hydrogen bond. Probability isocontour of 1 Å^−2^ is drawn. Results of CPMD and PIMD simulations of the n-octanol dimer in the gas phase.

Path integral MD simulations are not aimed at reproduction of dynamical properties, but rather statistical averages [62,63]. Hence, Figure 12 shows probability isocontour for the bridged proton position during the CPMD and PIMD runs, allowing for easy observation of the influence of nuclear effects on the molecular structure. The simulations were carried out in the gas phase for the n-octanol dimer. For the CPMD run, the analyzed part of the trajectory covered the period in which rotation of the hydroxyl groups was not registered (see Figure 8). According to the CPMD results the O1…O2 interatomic distance covers the range between 2.6 Å to 3.8 Å. The O-H distance was found to be between 0.92 Å–1.04 Å. Whereas, the PIMD results have drawn a following picture: the O1…O2 interatomic distance ranges from 2.5 Å to 3.3 Å and the O-H distance has been spread between ca. 0.85 Å and 1.16 Å. The comparison of the two methods showed that the inclusion of quantum effects in the nuclear dynamics affects the whole intermolecular hydrogen bond. In the CPMD description the O1…O2 interatomic distance is longer than in the PIMD. An opposite situation is observed for the OH distance where the inclusion of quantum effects elongated the distance indicating a stronger delocalization of the bridged proton. This result is related to the fact that proton position closer to the midpoint of the bond, thus more flat potential approaching the symmetric single well case, signifies stronger—and shorter—bridges. The reverse is also true: when the proton is more delocalized (in our case, by inclusion of nuclear quantum effects), the donor-acceptor separation tends to decrease. However, the inclusion of quantum effects confirmed that the proton transfer phenomena do not occur and the bridged proton is localized on the donor side.

### 3.7. A Posteriori Inclusion of Quantum Corrections to the Nuclear Motion—Isotope Effect

**Figure 13:**
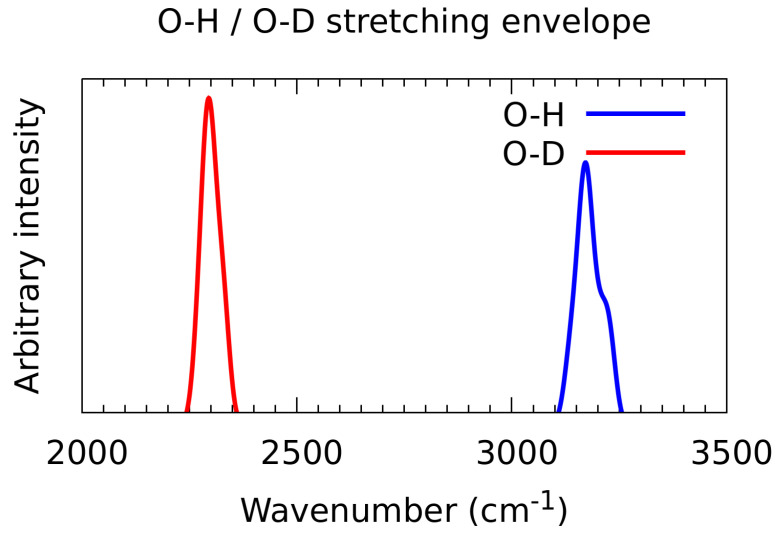
Envelope of the hydroxyl O-H/O-D stretching vibrational band-results of a posteriori quantization of nuclear motion with snapshot-envelope approach on the basis of CPMD simulation of the n-octanol trimer in the gas phase.

While the Path Integral MD approach allowed for description of the structural implications of the quantum nuclear motions, other approaches are needed to reproduce quantum corrections to the vibrational features. The snapshot-envelope technique [64] offers an interesting route to achieve this goal: it is calculated a posteriori, therefore isotope effects can be studied with the same dynamics trajectory by changing the particle mass from proton to deuteron at the vibrational wavefunction calculation stage. Moreover, a selected mode is quantized. In our case the O-H and O-D stretching modes were treated with this method, and the results are shown in Figure 13. The model used in this section is the n-octanol trimer in the gas phase, and the studied bridge, O2-H2…O3, was selected because it is most similar to the situation in the liquid phase. The O2 oxygen atom is both a donor and an acceptor of hydrogen bonds throughout the whole CPMD run (see Figure 9a,c). The O-H stretching band, spanning from 3100 to 3250 cm^−1^, exhibits a shoulder at 3220 cm^−1^, with its absorption maximum at 3160 cm^−1^. The O-D band covering the 2250–2360 cm^−1^ range, peaks at 2290 cm^−1^. This yields the isotope shift ratio of 1.38, less than the ideal harmonic ratio of 1.414, but more than the experimental value. Comparison of the simulated hydrogen-bonded νOH location within classical nuclear motion (Figure 11) and within snapshot-envelope approach (Figure 13) shows that an additional red shift due to the quantization of nuclear motions is ca. 100 cm^−1^.

Results of the quantum effects inclusions from the snapshot-envelope method give lower-wavenumber νOD location than experiment. This is partially caused by the snapshot-envelope intrinsic feature of relying on the CPMD trajectory, which was carried out with the O-H system. Thus, dynamical couplings might be different than in the experiment, affecting the explored phase space regions. Moreover, the CPMD simulations of vibrational features were carried out in the gas phase models, adding to the possible sources of less than perfect agreement with liquid phase experiment.

## 4. Conclusions

Intermolecular interactions and spectroscopic (FTIR) signatures in n-octanol have been investigated based on experimental and theoretical methods. The obtained data enabled detailed analyses of n-octanol molecular properties in the gas and liquid phases. The isotope effect was taken into consideration in the experimental as well as theoretical study. The experimental FTIR results gave an insight into main spectroscopic differences introduced by the isotope effect. The νOH band is located above the νCH region around 3300 cm${}^{-1}$ while for the deuterated form it is located below the νCH region—closer to 2600 cm${}^{-1}$. The harmonic spectra computed at the PBE/aug-cc-pVTZ level of theory are in good agreement with the experimental data. They are slightly shifted towards higher wavenumbers. The comparison of the X-ray and computed selected metric parameters, mainly associated with the intermolecular hydrogen bond, indicated that the chosen theoretical approach was able to reproduce correctly the chemical structure of the n-octanol monomer, dimer and trimer. The AIM analysis showed the electron density changes in the atoms involved in the intermolecular hydrogen bond formation. Moreover, the topological analysis confirmed the presence of the hydrogen bonding and other intermolecular interactions (e.g., C-H…O and C-H…H-C). The SAPT analysis revealed that n-octanol exhibits an unusual balance between two different types of interaction. It was found that, although dispersion is important also for the strength of hydrogen bonding, the aliphatic tail is long enough to provide significant additional dispersion interactions with similar hydrophobic neighbours. It is worth noticing that the strength of these interactions is comparable to the strength of the intermolecular hydrogen bonding established between hydroxyl groups. The classical MD simulations enabled us to draw conclusion concerning the dynamical nature of the intermolecular hydrogen bonds. According to the obtained results it was possible to make an assumption that the hydrogen bonds continually break and form again during the simulation time. The hydroxyl group is saturated with hydrogen bonds (two, based on the RDF profile), but relatively quickly the actual proton-donor and acceptor molecules can change due to the diffusion of n-octanol molecules in the liquid phase. The CPMD simulations provided data of the metric parameters of atoms involved in the intermolecular hydrogen bonds in the gas phase and in the liquid. The proton transfer phenomena are not preferable in the discussed hydrogen bonds. The computed, based on DFT and CPMD, spectroscopic properties have been well reproduced comparing to the experimental FTIR data. The inclusion of nuclear quantum effects revealed stronger delocalization of the bridged proton in the hydrogen bond. An application of the a posteriori quantization allowed the study of the quantum corrections to the vibrational features. The O2-H2…O3 intermolecular hydrogen bond was selected, because it was found to be the most similar to the situation in the liquid phase. The isotope effect was investigated as well. It was found that the simulated hydrogen-bonded νOH location derived from the CPMD and within snapshot-envelope method exhibited an additional red shift of ca. 100 cm^−1^.

## Figures and Tables

**Table 1:** Band assignment in liquid n-octanol and dueterated n-octanol spectra: comparison of experimental and computed data. The simulations were performed at the PBE/aug-cc-pVTZ with solvent reaction field (PCM and octanol as a solvent). Band positions are given in cm${}^{-1}$.

Literature Assignment	Experimental	Theoretical (in n-Octanol Dimer)
[16,17,98,99,100,101]		
stretching OH	3319	3698, 3387
stretching CH	2955, 2926	3025, 3018, 2998, 2990, 2983
	2871, 2855	2954, 2951, 2946, 2936, 2886
bending OH	1420, 1120	1424, 1180
stretching OD	2490	2700, 2537
scissoring CH${}_{2}$	1467	1462, 1451
twisting CH${}_{2}$	1378	1252
stretching C-O	1058, 989, 954	1038, 1020, 1005
and C-C		975, 961
rocking CH${}_{2}$	722	706, 702, 701

**Table 2:** AIM atomic charges for the n-octanol monomer, dimer and trimer computed at the PBE/aug-cc-pVTZ level of theory in the gas phase and with solvent reaction field.

Atom	Atomic Charge [e]					
	**Monomer**		**Dimer**		**Trimer**	
	**Gas Phase**	**PCM**	**Gas Phase**	**PCM**	**Gas** **Phase**	**PCM**
qH1	0.5437	0.5423	0.5834	0.5845	0.5922	0.5944
qO1	−1.0562	−1.0503	−1.1092	−1.1052	−1.1217	−1.1048
qC1	0.4688	0.4625	0.4891	0.4839	0.4931	0.4909
qH2			0.5560	0.5568	0.5982	0.5958
qO2			−1.0588	−1.0553	−1.1145	−1.1077
qC2			0.4457	0.4434	0.4702	0.4635
qH3					0.5561	0.5592
qO3					−1.0595	−1.0582
qC3					0.4247	0.4366

**Table 3:** Bond Critical Points (BCPs) obtained for the selected geometric parameters of n-octanol dimer and trimer calculated at the PBE/aug-cc-pVTZ level of theory in the gas phase and with solvent reaction field. Electron density $\rho_{BCP}$ is given in $e\cdot a_{0}^{-3}$ atomic units, and its Laplacian $\nabla^{2}\rho_{BCP}$ in $e\cdot a_{0}^{-5}$ units.

**Dimer**				
	**Gas phase**		**PCM**	
***BCP***	$\rho_{\mathrm{BCP}}$	$\nabla^{2}\rho_{\mathrm{BCP}}$	$\rho_{\mathrm{BCP}}$	$\nabla^{2}\rho_{\mathrm{BCP}}$
C1-O1	0.2585	−0.5887	0.2564	−0.5916
O1-H1	0.3503	−2.4583	0.3458	−2.4082
H1…O2	0.0310	0.0830	0.0351	0.0894
O2-C2	0.2443	−0.5164	0.2431	−0.5165
**Trimer**				
	**Gas phase**		**PCM**	
***BCP***	$\rho_{\mathrm{BCP}}$	$\nabla^{2}\rho_{\mathrm{BCP}}$	$\rho_{\mathrm{BCP}}$	$\nabla^{2}\rho_{\mathrm{BCP}}$
C1-O1	0.2596	−0.5915	0.2595	−0.6138
O1-H1	0.3425	−2.4002	0.3407	−2.3819
H1…O2	0.0373	0.0882	0.0383	0.0897
O2-C2	0.2522	−0.5533	0.2495	−0.5516
O2-H2	0.3413	−2.4018	0.3401	−2.3802
H2…O3	0.0384	0.0884	0.0392	0.0904
O3-C3	0.2385	−0.4912	0.2412	−0.5044

**Table 4:** SAPT partitioning of the interaction energy in the n-octanol dimers. All data in kcal/mol.

	X-ray Structures			DFT-Optimized Structures		
SAPT Term	Dimer 1	Dimer 2	Dimer 3	Dimer 1	Dimer 2	Dimer 3
Electrostatics	−7.99	−1.55	−1.04	−10.64	−0.60	−1.18
Exchange	9.44	3.55	4.60	12.37	1.64	3.54
Induction	−2.56	−0.22	−0.42	−3.85	−0.14	−0.40
Dispersion	−3.27	−5.21	−7.93	−3.76	−3.10	−6.59
Total SAPT2	−4.39	−3.43	−4.79	−5.87	−2.21	−4.62

## Data Availability

The data relevant to this article are contained within the article itself and in the Supplementary Material File.

## Supplementary Materials

Table of contents: Figure S1. Atoms numbering scheme used in Tables S1 and S2. Atoms coloring scheme: oxygen atom—red, hydrogen atoms—white and carbon atoms—grey. The numbering scheme of selected atoms was prepared especially for the current study. The presence of the intermolecular hydrogen bond is denoted by dotted line. Only the hydrogen atom from the hydroxyl group was deuterated (H/D). Figure S2. Calculated IR spectra of n-octanol monomer. The simulations were performed at the PBE/aug-cc-pVTZ level of theory in the gas phase (GP) and with solvent reaction field reproduced by PCM model. D—denotes deuter. Figure S3. Calculated IR spectra of n-octanol dimer. The simulations were performed at the PBE/aug-cc-pVTZ level of theory in the gas phase (GP) and with solvent reaction field reproduced by PCM model. D—denotes deuter. Figure S4. Calculated IR spectra of n-octanol trimer. The simulations were performed at the PBE/aug-cc-pVTZ level of theory in the gas phase (GP) and with solvent reaction field reproduced by PCM model. D—denotes deuter. Table S1. Selected bond lengths in n-octanol—comparison of X-ray data [113] with density functional theory (DFT) results. The simulations were performed at the PBE/aug-cc-pVTZ level of theory in the gas phase and with solvent reaction field (PCM model and octanol as a solvent). Table S2. Selected valence angles in n-octanol—comparison of X-ray data [113] with density functional theory (DFT) results. The simulations were performed at the PBE/aug-cc-pVTZ level of theory in the gas phase and with solvent reaction field (PCM model and octanol as a solvent). Table S3. Location and intensity of νOH/ νOD band from DFT calculated spectra of different n-octanol systems. The simulations were performed at the PBE/aug-cc-pVTZ level of theory in the gas phase and with solvent reaction field (PCM model and octanol as a solvent).

## Abbreviations

The following abbreviations are used in this manuscript:

FTIR	Fourier Transform Infrared Spectroscopy
DFT	Density Functional Theory
HB	hydrogen bond
PCM	Polarizable Continuum Model
AIM	Atoms In Molecules
MD	Molecular Dynamics
CPMD	Car-Parrinello Molecular Dynamics
PIMD	Path Integral Molecular Dynamics
NDE	Nonlinear Dielectric Effect
FPMD	First Principle Molecular Dynamics
CCDC	Cambridge Crystallographic Data Centre
PES	Potential Energy Surface
BCP	Bond Critical Point
RCP	Ring Critical Point
CCP	Cage Critical Point
SAPT	Symmetry-Adapted Perturbation Theory
BSSE	Basis Set Superposition Error
MP2	Møller-Plesset second-order perturbation theory
PME	particle mesh Ewald
PBC	Periodic Boundary Conditions
RMSD	Root Mean Square Deviation
RDF	Radial Distribution Function
