# Supplementary material for: Intermolecular Interactions and Spectroscopic Signatures of the Hydrogen-Bonded System—n-Octanol in Experimental and Theoretical Studies
Source: Molecules. 2022 Feb 11;27(4):1225. doi: 10.3390/molecules27041225 (PMC8878718; doi:10.3390/molecules27041225)
Supplement: Supplementary file 1 [file molecules-27-01225-s001.zip › molecules-1577503-supplementary.pdf]

SUPPLEMENTARY MATERIAL

Intermolecular interactions and spectroscopic signatures of the hydrogen-bonded system - n-octanol in experimental and theoretical studies

Michał Pocheć[1], Katarzyna M. Krupka[1], Jarosław J. Panek[1], Kazimierz Orzechowski[1] and Aneta Jezierska[1]**

[1]University of Wrocław, Faculty of Chemistry, ul. F. Joliot-Curie 14, 50-383 Wrocław, Poland

Correspondence should be addressed to: michal.pochec@chem.uni.wroc.pl, aneta.jezierska@chem.uni.wroc.pl;
tel.: +48 71 3757 224; fax: +48 71 3282 348

Table of contents:

I. **Figure S1.** Atoms numbering scheme used in Tables S1 and S2. Atoms coloring scheme: oxygen atom - red, hydrogen atoms - white and carbon atoms - grey. The numbering scheme of selected atoms was prepared especially for the current study. The presence of the exemplary intermolecular hydrogen bond is denoted by dotted line. Only the hydrogen atom from the hydroxyl group was deuterated (H/D).

II. **Figure S2.** Calculated IR spectra of n-octanol monomer. The simulations were performed at the PBE/aug-cc-pVTZ level of theory in the gas phase (GP) and with solvent reaction field reproduced by PCM model. D – denotes deuter.

III. **Figure S3.** Calculated IR spectra of n-octanol dimer. The simulations were performed at the PBE/aug-cc-pVTZ level of theory in the gas phase (GP) and with solvent reaction field reproduced by PCM model. D – denotes deuter.

IV. **Figure S3.** Calculated IR spectra of n-octanol trimer. The simulations were performed at the PBE/aug-cc-pVTZ level of theory in the gas phase (GP) and with solvent reaction field reproduced by PCM model. D – denotes deuter.

V. **Table S1.** Selected bond lengths in n-octanol – comparison of X-ray data [1] with density functional theory (DFT) results. The simulations were performed at the PBE/aug-cc-pVTZ level of theory in the gas phase and with solvent reaction field (PCM model and octanol as a solvent).

VI. **Table S2.** Selected valence angles in n-octanol – comparison of X-ray data [1] with density functional theory (DFT) results. The simulations were performed at the PBE/aug-cc-pVTZ level of theory in the gas phase and with solvent reaction field (PCM model and octanol as a solvent).

VII. **Table S3.** Location and intensity of νOH/ νOD band from DFT calculated spectra of different n-octanol systems. The simulations were performed at the PBE/aug-cc-pVTZ level of theory in the gas phase and with solvent reaction field (PCM model and octanol as a solvent).

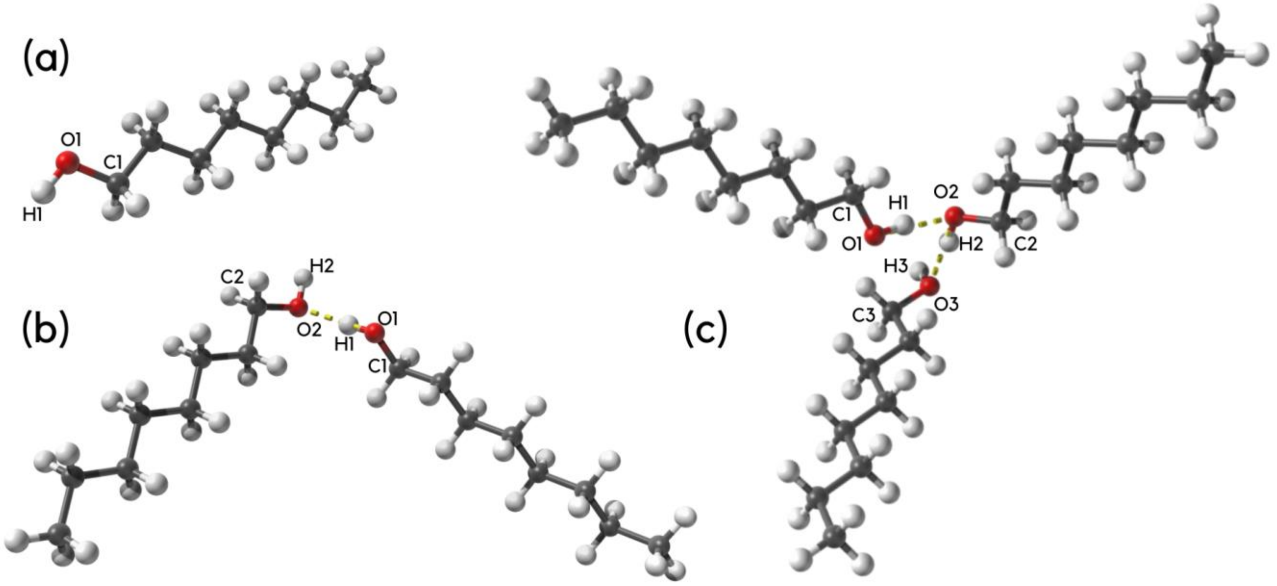

Figure S1. Atoms numbering scheme used in Tables S1 and S2. Atoms coloring scheme: oxygen atom - red, hydrogen atoms - white and carbon atoms - grey. The numbering scheme of selected atoms was prepared especially for the current study. The presence of the exemplary intermolecular hydrogen bond is denoted by dotted line. Only the hydrogen atom from the hydroxyl group was deuterated (H/D).

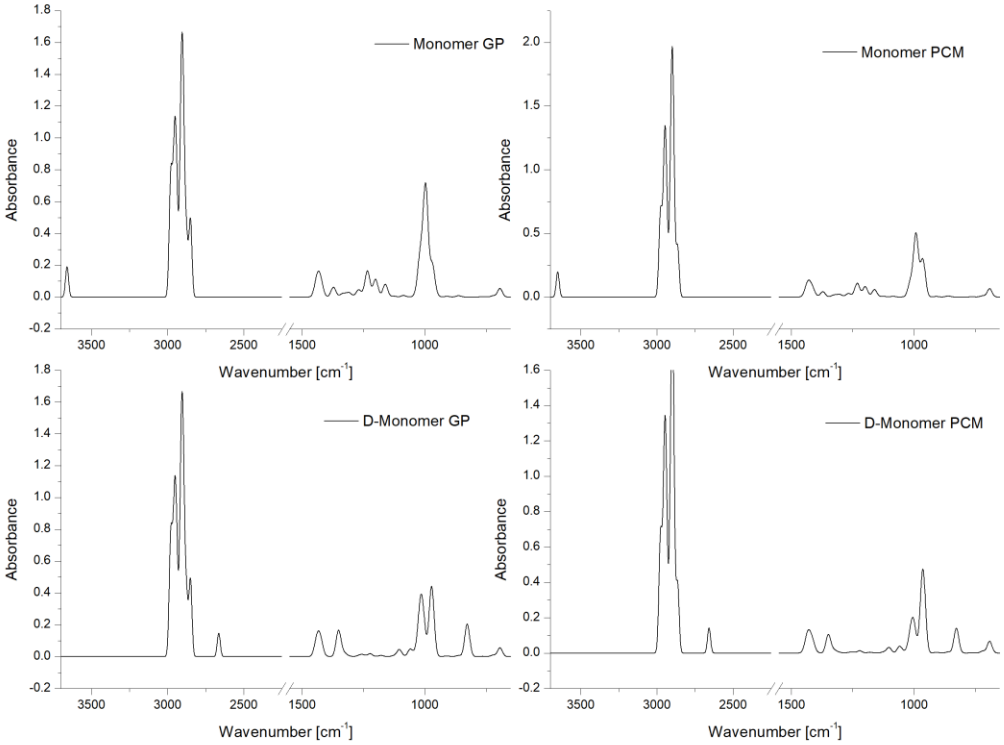

Figure S2. Calculated IR spectra of n-octanol monomer. The simulations were performed at the PBE/aug-cc-pVTZ level of theory in the gas phase (GP) and with solvent reaction field reproduced by PCM model. D – denotes deuter.

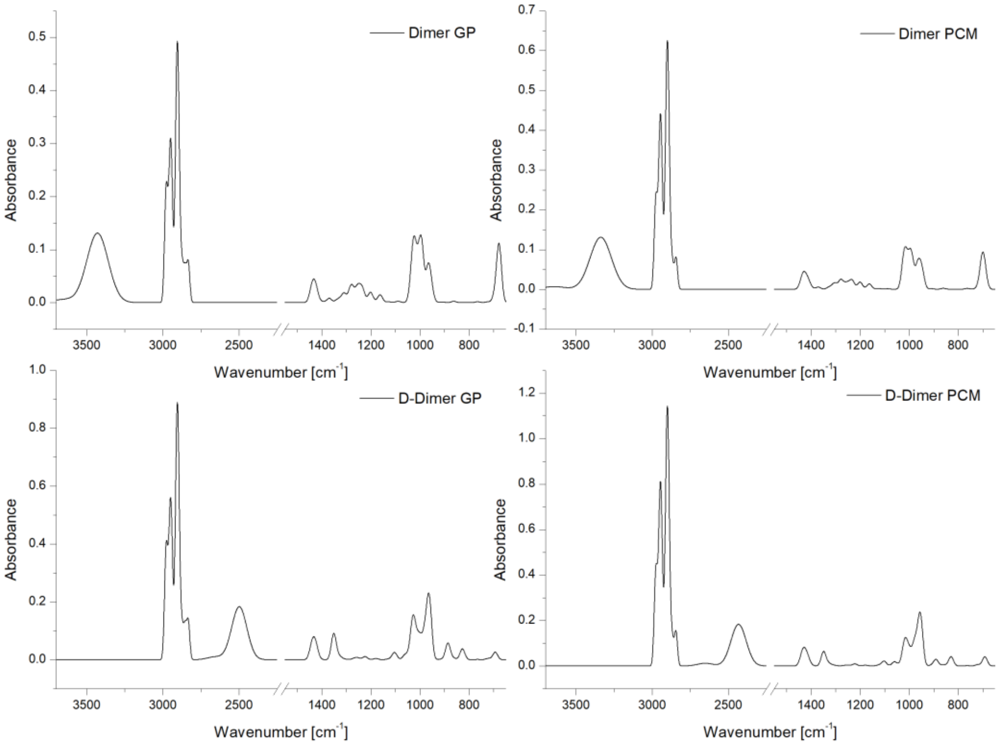

Figure S3. Calculated IR spectra of n-octanol dimer. The simulations were performed at the PBE/aug-cc-pVTZ level of theory in the gas phase (GP) and with solvent reaction field reproduced by PCM model. D – denotes deuter.

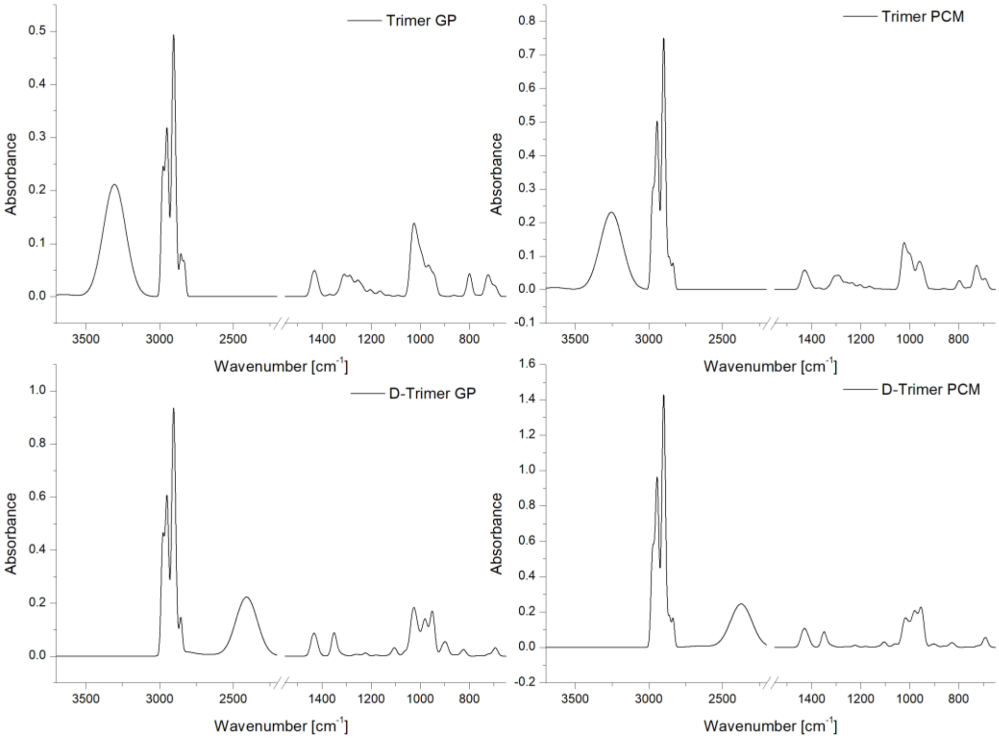

Figure S4. Calculated IR spectra of n-octanol trimer. The simulations were performed at the PBE/aug-cc-pVTZ level of theory in the gas phase (GP) and with solvent reaction field reproduced by PCM model. D – denotes deuter.

Table S1. Selected bond lengths in n-octanol – comparison of X-ray data [1] with density functional theory (DFT) results. The simulations were performed at the PBE/aug-cc-pVTZ level of theory in the gas phase and with solvent reaction field (PCM model and octanol as a solvent).

System	Method	C-O1	O1-H1	H1...O2	O1...O2	O2-C	O2-H2	H2...O3	O2...O3	O3-C	O3-H3
		[Å]	[Å]	[Å]	[Å]	[Å]	[Å]	[Å]	[Å]	[Å]	[Å]
Monomer	Gas Phase	1.436	0.970	-	-	-	-	-	-	-	-
	PCM	1.441	0.971	-	-	-	-	-	-	-	-
Dimer	Gas Phase	1.427	0.982	1.886	2.864	1.446	0.971	-	-	-	-
	PCM	1.432	0.986	1.831	2.817	1.449	0.972	-	-	-	-
Trimer	Gas Phase	1.425	0.988	1.814	2.791	1.435	0.989	1.803	2.784	1.455	0.971
	PCM	1.429	0.991	1.798	2.789	1.440	0.991	1.789	2.780	1.451	0.972
Experimental (X-Ray) [1]		1.435	0.901	1.924	2.821	1.435	0.901	1.924	2.821	1.435	0.901

Table S2. Selected valence angles in n-octanol – comparison of X-ray data [1] with density functional theory (DFT) results. The simulations were performed at the PBE/aug-cc-pVTZ level of theory in the gas phase and with solvent reaction field (PCM model and octanol as a solvent).

System	Method	C-O1-H1	O1-H1-O2	H1-O2-C	H1-O2-H2	O2-H2-O3	H2-O3-C	H2-O3-H3
		[°]	[°]	[°]	[°]	[°]	[°]	[°]
Monomer	Gas Phase	108.1	-	-	-	-	-	-
	PCM	107.7	-	-	-	-	-	-
Dimer	Gas Phase	108.3	173.9	112.4	108.7	-	-	-
	PCM	107.9	178.7	117.1	108.7	-	-	-
Trimer	Gas Phase	108.7	169.1	118.4	99.0	170.5	110.4	106.7
	PCM	107.5	179.0	115.7	113.2	177.4	115.5	107.1
Experimental (X-Ray) [1]		104.4	173.7	119.8	133.7	173.7	119.8	133.7

Table S3. Location and intensity of νOH/ νOD band from DFT calculated spectra of different n-octanol systems. The simulations were performed at the PBE/aug-cc-pVTZ level of theory in the gas phase and with solvent reaction field (PCM model and octanol as a solvent).

System	Method	νOH/ νOD [cm^{-1}]	I $_{\nu OH/\nu OD}$ [km/mol]
Monomer	Gas Phase	3716,33	20,738
	PCM	3706,03	38,107
	Deuterated Gas Phase	2705,60	15,946
	Deuterated PCM	2698,05	27,488
Dimer	Gas Phase	3481,87<br>3708,87	729,274<br>31,487
	PCM	3387,87<br>3698,38	1053,448<br>55,320
	Deuterated Gas Phase	2537,21<br>2700,07	403,873<br>21,497
	Deuterated PCM	2469,65<br>2692,46	572,963<br>36,878
Trimer	Gas Phase	3323,43<br>3377,91<br>3706,05	789,882<br>1076,943<br>32,158
	PCM	3279,01<br>3332,14<br>3694,91	1317,145<br>1170,876<br>59,906
	Deuterated Gas Phase	2424,97<br>2461,25<br>2697,91	434,483<br>573,921<br>21,698
	Deuterated PCM	2392,21<br>2428,79<br>2690,00	688,263<br>645,515<br>39,497

References:

[1] Howard A. Shallard-Brown, David J. Watkin, Andrew R. Cowley, n-Octanol. *Acta Crystallogr. E*, **2005**, *61*, o213-o214.
